# Sleep in adults with autism spectrum disorder and adhd: A meta-analysis

**DOI:** 10.1192/j.eurpsy.2021.454

**Published:** 2021-08-13

**Authors:** J.A. Ramos-Quiroga

**Affiliations:** Department Of Psychiatry, Hospital Universitari Vall d’Hebron. Universitat Autònoma de Barcelona, Barcelona, Spain

**Keywords:** meta-analysis, ADHD, autism, sleep

## Abstract

**Introduction:**

Sleep-related problems have been frequently reported in neurodevelopmental disorders, with special emphasis in Autism Spectrum Disorder (ASD) and Attention Deficit/Hyperactivity Disor-der (ADHD).

**Objectives:**

To perform a meta-analysis (PROSPERO’s CRD42019132916) on sleep disturbances in adults with ASD and/or ADHD.

**Methods:**

A total of 1126 studies and 66 references were identified by electronic and manual searches, respectively. Of these, 42 studies were included in the meta-analysis.

**Results:**

showed that both disorders share a similar sleep-impaired profile with higher sleep onset latency, poorer sleep efficiency, greater number of awakenings during sleep, and a general lower self-perceived sleep quality compared with healthy controls. A higher proportion of N1 sleep was found in ASD participants, while a greater Periodic Limb Movements in Sleep is specific in ADHD adults.

**Conclusions:**

Sleep is impaired by several sleep problems and disorders in both ASD and ADHD adults. More research is needed to develop more awareness in mental healthcare, and better treatment of this impairing comorbidity in ASD and ADHD

**Disclosure:**

No significant relationships.

